# Comparison of the Clinical Evaluation of Digital Tooth Shade Determination Using an Intraoral Scanner with Proven Subjective and Objective Methods

**DOI:** 10.3390/jcm13226668

**Published:** 2024-11-06

**Authors:** Nicolai Budde, Christin Arnold, Andreas Wienke, Ramona Schweyen

**Affiliations:** 1 Department of Prosthodontics, Martin-Luther-University Halle-Wittenberg, Magdeburger Str. 16, 06112 Halle (Saale), Germany; christin.arnold@uk-halle.de (C.A.); ramona.schweyen@uk-halle.de (R.S.); 2 Institute of Medical Epidemiology, Biometry and Computer Science, Martin-Luther-University Halle-Wittenberg, Magdeburger Str. 8, 06112 Halle (Saale), Germany; andreas.wienke@uk-halle.de

**Keywords:** color perception, dental esthetics, dental technology, oral diagnosis, reproducibility of results

## Abstract

**Background/Objectives**: Despite the increasing use of chromatic intraoral scanners, color determination is often performed visually, offering poor reliability and validity. In this study, we aimed to compare the reliability and validity of the tooth shade determination tool of an intraoral scanner (Trios3 Color) with that of two dental spectrophotometers (VITA Easyshade Advance 4.0 and SpectroShade Micro) and with visual determination using the VITA 3D-Master shade guide. **Methods**: In vivo tooth shade determination was performed on 33 participants using positioning splints for the right central incisor. Repeated measurements assessed the reliability in determining the percentage agreement with VITA 3D-Master shades. VITA Easyshade measurements were used as reference values for validity. The metric value Delta E (ΔE) in the International Commission on Illumination L*a*b* color space was compared to the reference, with ΔE greater than 1 indicating visible differences and a maximum value of ΔE 6.8 being clinically acceptable. **Results**: The reliabilities of VITA Easyshade, the intraoral scanner, and SpectroShade Micro were 75.8%, 87.9%, and 89.9%, respectively. The visual method had an agreement rate of 20.6%. Validity values, compared with the reference value, were ΔE 3.8 (clinically acceptable), 8.3, and 7.4 (the latter two both being clinically unacceptable) for the intraoral scanner, area-measuring device, and visual method, respectively. **Conclusions**: The intraoral scanner is a reliable and valid tool for tooth shade determination and is superior to the visual method in both aspects.

## 1. Introduction

Intraoral scanners for dental impressions have become much more widespread in recent years. The accurate transfer of the intraoral situation is essential for precise prosthetic work. While full-arch impressions still rely somewhat on analog processes, partial impressions yield highly precise results [1]. The advantages of digital processes, such as fewer work steps and reduced sources of error, are clear. Furthermore, treatment comfort is improved, time is saved, and difficult situations (scanning divergent implants directly during surgery for temporary restorations) are managed more easily [2,3,4]. However, intraoral scanners have limitations in capturing edentulous jaw areas, moist surfaces, and functional impressions for complete dentures [5]. These limitations can be overlooked when determining tooth shade.

Visual tooth shade determination varies both intra- and inter-individually due to several physiological and physical factors [6,7,8], and dental shade measuring devices are used to limit variability [9]. The integration of standardized light sources and detectors helps maintain consistent conditions and eliminate harmful exogenous factors [10]. Digital methods are superior to visual methods for tooth shade determination [11,12,13,14,15,16,17].

Colorimeters, spectrophotometers, and digital cameras for visual comparisons have been used to electronically determine tooth shade [18]. Spectrophotometers analyze the full spectrum of reflected light and its radiant intensities using spectral filters that split visible light at intervals of 1–25 nm [18]. Spectrophotometers are more expensive; however, they are superior to colorimeters [6,19,20,21,22,23]. Point-measuring devices, which measure the color of a three- to five-millimeter section of the tooth surface in contact with the measuring tip, differ from area-measuring devices, which capture a digital image of the entire tooth [18]. However, point-measuring devices are prone to the edge loss effect, where brightness is lost at the measuring edge due to light scattering [24]. Area-measuring devices, on the other hand, lose accuracy due to software interpolation, which calculates an arithmetic mean value from a larger area [22]. Intraoral scanners allow digital data processing via point-by-point scanning of an object using light or lasers in stripes or areas [2]. Chromatic intraoral scanners detect color using the spectral composition of the light source, projecting different wavelengths [2].

Despite its limitations, the visual method remains the standard for determining tooth color, as digital tooth color determination is often not taught in dental schools [25,26]. Intraoral scanners combine impression making and tooth shade determination, and their clinical use for determining tooth color could therefore become more widespread.

The three-dimensional CIELAB color system, which uses the coordinates L*, a*, and b*, developed by the International Commission on Illumination (CIE; Commission Internationale de la Éclairage, French) in 1976, serves as an international standard [6]. The vertical L* axis represents brightness, with gray values ranging from black (0) to white (100). The a* axis is the green–red coordinate (negative a* for green, positive a* for red), and the b* axis is the blue–yellow coordinate (negative b* for blue, positive b* for yellow) [6]. This system allows each color to be pinpointed in three-dimensional space. The difference between two colors in the CIELAB model is described as the Euclidean distance between two points in three-dimensional space, using the following formula [19]:

$$\Delta E=\sqrt{{\left(L1-L2\right)}^{2}+{\left(a1-a2\right)}^{2}+{\left(b1-b2\right)}^{2}}$$

The mathematical calculations of color distance in the CIELAB model have been optimized with more complex formulas; this Delta E (ΔE) 76 formula remains commonly used in publications [25]. This may be attributed to its comparability with other studies, thanks to its long-term use and the simple interpretation of color differences based on established threshold values. At these thresholds, 50% of participants could detect a color difference (visibility) or deem the difference unacceptable for clinical use (clinical acceptability) [27]. A color difference of ΔE = 1 can be perceived by the human eye under laboratory conditions [28,29]. However, clinical acceptability is in the ΔE = 2.72–6.8 range, depending on the examination conditions [30].

Methods for determining tooth shade are evaluated based on two quality criteria: validity and reliability [19,31]. Validity refers to the accuracy of a measurement or its agreement with a reference value, while reliability refers to the consistency of multiple measurements. Inaccurate results may occur due to device or application errors.

Numerous studies have investigated the validity and reliability of devices used for tooth shade determination [32,33]. Spectrophotometers with point-measurement devices are often considered the reference standard due to their high validity and reliability [10,22,30,34]. However, the devices examined vary due to the rapid pace of digitization. Moreover, thresholds for visibility and clinical acceptability differ, and many previous studies were conducted only in vitro, with different reference devices used for validity comparisons. To better understand trends in functionality and performance among measurement methods, further studies involving multiple tooth shade determination devices are needed. Therefore, in this study, we aimed to evaluate the validity and reliability of the tooth shade determination tool of an intraoral scanner and compare it with visual and spectrophotometric methods. The primary focus of our research was on validity comparisons with a reference system. The null hypothesis was that comparing individual tooth shade determination methods with a reference would not yield differences of ΔE > 1, indicating that the intraoral scanner software could provide an equivalent tooth shade determination mechanism.

## 2. Materials and Methods

This study was conducted in 2020 at the university’s clinic for prosthodontics. The study protocol was approved by the university’s ethics committee (No. 2018-183, approval date 01.07.2019) and is in accordance with the principles of the Declaration of Helsinki.

**Figure 1:**
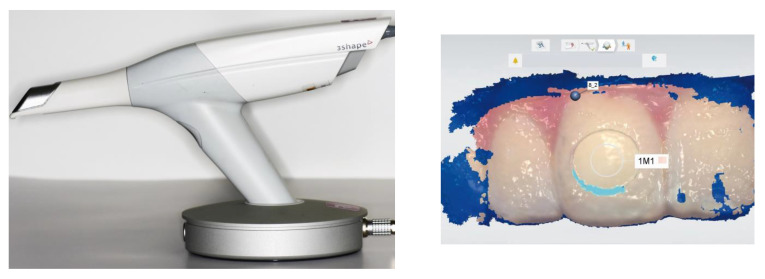
Side view of T and second measurement screen for participant no. 8 with tooth shade determined as 1M1. T = intraoral scanner.

The validity and reliability of various objective tooth shade determination systems (Table 1) were compared under clinical conditions with visual tooth shade determination (daylight, north-facing window, participants seated on a dental treatment unit). The devices used included a chromatic intraoral scanner (TRIOS3 Color Portable Operating Device POD, 3Shape, Copenhagen, Denmark; T; Figure 1), a spectrophotometer with a point-measuring device (VITA Easyshade Advance 4.0, VITA Zahnfabrik, Bad Saeckingen, Germany; V), and a spectrophotometer with a digital color imaging system as an area-measuring device (SpectroShade Micro, MHT s.r.l. a socio unico, Verona, Italy; S). To account for the influence of experience on visual tooth shade determination, experienced dental technicians (DTs) determined the tooth shade visually [6] using the VITA 3D-Master shade guide (VITA Zahnfabrik, Bad Saeckingen, Germany). These instruments were selected based on their frequent mentions in the literature [2,10,22,30,34,35]. According to Hack and Patzelt [36], the TRIOS3 intraoral scanner demonstrated the best results for validity and reliability compared to five other systems.

A preliminary in vitro study was conducted to establish a reference standard, followed by a clinical evaluation of the four tooth shade determination methods.

Since both the TRIOS3 Color POD (T) and the visual method performed by DTs only provide VITA 3D-Master shades without corresponding CIE L*a*b values, a conversion table was created (Table 2). Twenty-nine shades from the VITA 3D-Master shade guide were measured five times using the VITA Easyshade Advance (V), and the arithmetic mean values were calculated to generate the conversion table.

Dental students meeting the following inclusion criteria were enrolled in this study: aged ≥ 18 years, healthy vital upper right central incisors without restorations, no active periodontal disease, and no history of smoking. Exclusion criteria included prior conservative or prosthetic treatment of the upper incisors, untreated or uncontrolled caries, active periodontal disease, and smoking. Participation was voluntary, and written informed consent was obtained before the commencement of this study.

Based on a sample size analysis by Lenth [37], a *t*-test indicated the need for 27 participants to achieve 80% power, with a Bonferroni-corrected alpha of 1.67%. The target sample size was increased by 10% to account for potential dropouts, with the aim of including a minimum of 30 students.

**Figure 2:**
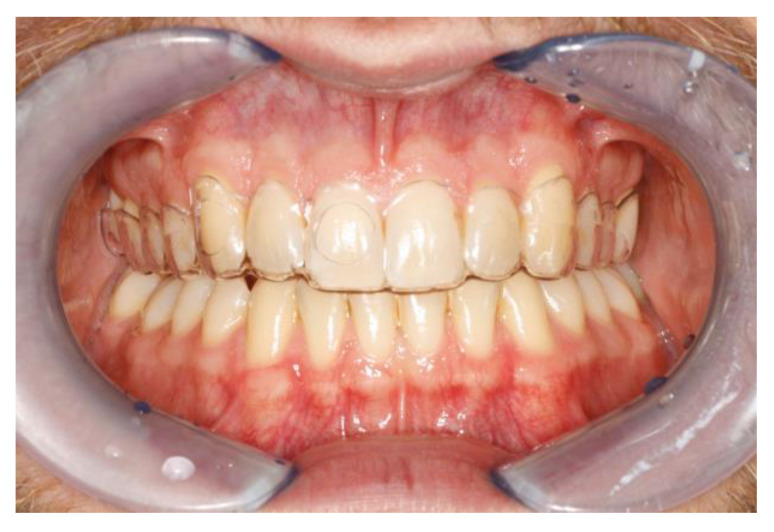
Positioning splint (additional measuring position at 13 for alternative study design).

The upper right central incisor was measured. Since a constant color temperature cannot be guaranteed by daylight alone, artificial light sources (OSRAM FQ HO 49W/865, Osram Licht AG, Munich, DE, with a color temperature of 6500 K) were used. A maxillary splint with a 5 mm diameter hole milled in the middle third of the upper right incisor was fabricated for each participant to standardize the measurement point (Erkodur, Erkodent Erich Kopp GmbH, Pfalzgrafenweiler, Germany; Figure 2).

**Figure 3:**
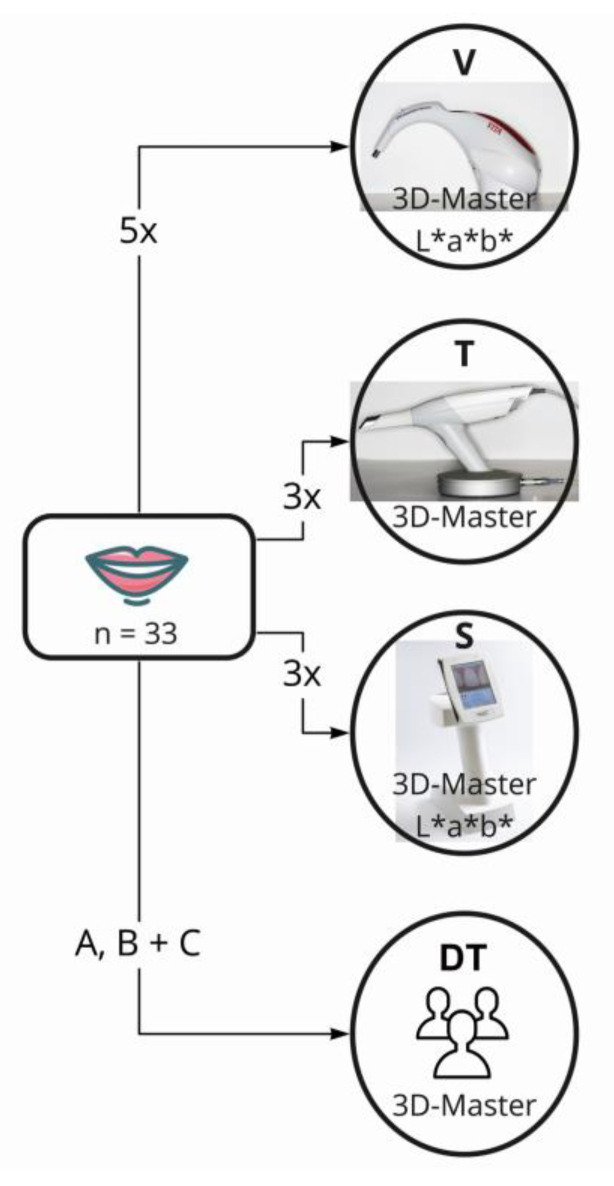
Scheme of measurements. n = number of test persons, measurement repetitions on arrows; A, B, and C = designation of three dental technicians; V = VITA Easyshade Advance 4.0; T = TRIOS3 Color Pod; S = SpectroShade Micro; DT = dental technician. Display of measured values within the circle as VITA 3D-Master color or CIE L*a*b* value.

The same dentist used three objective measurement methods in immediate succession (Table 1, Figure 3) to determine the tooth shade of each participant’s incisor. All devices were disinfected and calibrated before use, and measurements were performed according to the manufacturer’s instructions. Participants were instructed to rewet their teeth between measurements to prevent dehydration.

The measurements from V were used as the reference for validity, and five direct measurements were taken for each participant [10,22,30,34]. In contrast, three measurements were taken with the SpectroShade Micro (S) and T due to the longer time required to obtain the measurements in clinical settings. For T, a laboratory order must be created in the software for each scan, and a larger area needs to be scanned and recalculated. In clinical practice, tooth shade determination is only carried out once, in addition to scanning the preparation. Arithmetic mean values were calculated for all measurements. In addition, visual tooth shade determination was performed by three DTs to assess inter-rater reliability (reproducibility) (Figure 3). The absence of color vision deficiencies in the DTs was confirmed through anamnesis.

### Statistical Analysis

To assess reliability, three to five direct repeat measurements from the different devices were compared with those from the VITA 3D-Master shades as nominal data in comparative pairs, and the agreement (%) was calculated. This analysis was also conducted to determine the agreement rate among the three DTs (inter-rater reliability). Differences in agreement rates between methods were analyzed globally using Pearson’s chi-squared test, and pairwise comparisons were conducted post hoc using additional Pearson’s chi-squared tests. Fisher’s exact test was used to calculate the *p*-value when the expected frequency was <5. A *p*-value of ≤0.05 was considered statistically significant, indicating a difference that is not random. The effect size or strength of this test was determined using Cramer’s V, with small (V = 0.1), medium (V = 0.3), and large (V = 0.5) effects defined according to Cohen [38].

The CIE L*a*b* arithmetic mean values of the tooth shade determination methods were compared pairwise, and the Euclidean distance (ΔE) between two color points in the three-dimensional CIELAB color space was calculated. The primary research question focused on the in vivo validity of the three measurement methods—T, S, and visual assessments by DTs—compared with that of the reference method, V. A one-sample *t*-test was used to assess whether the tooth shade determination methods yielded visibly different colors at a test value of 1, based on the minimum visibility threshold. The *p*-value was calculated to determine the strength of the test results, with a *p*-value of ≤ 0.05 indicating a significant deviation from the test value of 1.

Statistical analyses were conducted using Microsoft Excel 2010 (Microsoft Corp., Redmond, Washington, DC, USA) and the Statistical Package for the Social Sciences Statistics platform (Version 25, IBM Corp., Armonk, NY, USA).

## 3. Results

The results of the preliminary in vitro shade measurements are presented in Table 2, which shows the conversion of VITA 3D-Master shades to CIE L*a*b values.

Table 3 displays the in vivo match rates (%) of the nominal VITA 3D-Master colors per measuring device. A tendency towards random differences was observed between the two methods (Table 4). T and S indicated weak effect size equivalence in percentages with only random differences. Therefore, a reliable difference could not be detected with the current sample size for the values of 87.9% (T) and 89.9% (S). The other percentages demonstrated detectable differences with small effect sizes.

The agreement rate of the nominal VITA 3D-Master colors among the three DTs (A, B, and C) was 20.6% (inter-rater reliability).

**Figure 4:**
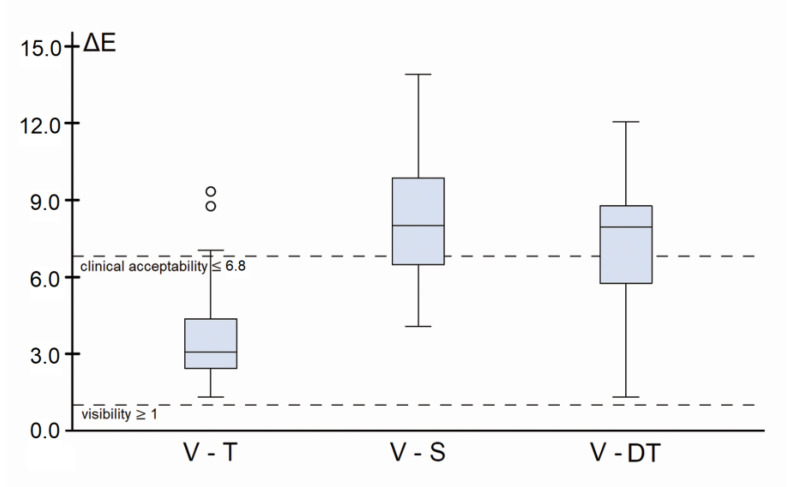
Boxplot of color difference in ΔE as validity. The threshold of visibility is shown as the lower limit at ΔE = 1. The threshold of clinical acceptability is defined as ΔE = 6.8 [30]. Outliers are marked as small circles.

The arithmetic mean values and standard deviations of ΔE in the in vivo validity comparisons related to the main research question were calculated (Table 5). The one-sample *t*-test, with the test level set at ΔE = 1, revealed statistically significant differences (*p*-values < 0.001), indicating that all three methods measured tooth colors that differed from the reference value. In each case, the difference exceeded the test value of 1 (the visibility threshold under laboratory conditions). The values obtained from one randomly selected DT (DT-B) were analyzed for the visual method. Figure 4 illustrates the color differences in ΔE. The S and visual method by DT exceeded the threshold for clinical acceptability (ΔE = 6.8) [30] in more than half of all measurements.

All methods demonstrated significant visible differences (*p* < 0.001; ΔE ≥ 1) in the validity comparisons, leading to the rejection of the null hypothesis. The colors determined by T were clinically acceptable (ΔE = 3.8 ± 2.0), relative to the upper limit of ΔE = 6.8. In contrast, the colors determined by S (ΔE = 8.3 ± 2.4) and the DTs (ΔE = 7.4 ± 2.6) were deemed unacceptable.

## 4. Discussion

A previous study [35] used an area-measuring device (SpectroShade Micro) with a conversion table as the reference system. In contrast, a Trios Color intraoral scanner (without version specification) yielded clinically acceptable values (ΔE = 3.4 ± 2.19) compared to the reference device (S) in this study. However, significant deviations (ΔE = 6.83 ± 4.44) were noted when compared to the values obtained by the point-measuring device (VITA Easyshade Advance 4.0), which served as the reference in this study.

The apparent superiority of the T in CIE L*a*b* value comparisons is influenced by the conversion table, which limits the color variability of T to just 29 possible CIE L*a*b* values. Devices with independently registered CIE L*a*b* values deviated significantly from this reference. Notably, visual tooth shade determination, both in this study and in Mehl et al. [35], did not benefit from the conversion table and exhibited greater deviation from the other measurements.

Therefore, despite the bias introduced by the conversion table, tooth shade determination using the intraoral scanner yields better results than the visual method. Several studies have suggested the superiority of intraoral scanners over visual methods [17,39,40].

The nominal agreement rate (device and application errors) for V was comparatively lower, at 75.8%. Despite the placement of the positioning splint, deviations occurred more frequently with the point-measuring device than with S (89.9%) or T (87.9%). Slight changes in the placement of the measuring point on the curved tooth surface, which can be considered an application error, likely reduced reliability. The area-measuring device benefited from internal angle control, ensuring consistent measurement quality. Similarly, T appeared to be more resilient to application errors when capturing the entire tooth. Furthermore, since the manufacturer did not disclose the internal algorithm used by the measuring devices to determine VITA 3D-Master shades, potential system errors remained undetected.

The gradation was larger between nominal colors (e.g., VITA 3D-Master) than in metric ΔE comparisons. Sarafianou et al. [23] reported a mean deviation of ΔE = 5 between two VITA 3D-Master shades, indicating that this difference can be clinically distinguished. Consequently, the “true” color may lie between two color schemes, potentially at the exact midpoint, where both colors are equally available for selection. If one system selects one color scheme while another chooses a different one, the difference is less accurately represented than in metric comparisons. Mehl et al. [35] referred to this phenomenon as “measurement noise” in terms of repeatability. The agreement rate of 20.6% for the nominal VITA 3D-Master shades among all three DTs indicated a very low, almost arbitrary reproducibility of the visual method.

The limitations of the ΔE metric value comparisons in this study stem from their purely quantitative nature, making it impossible to qualitatively assess the direction of the difference, such as whether colors are lighter or darker [30]. To maintain consistency, the same dentist operated all color-measuring devices; however, this could introduce examiner bias. Furthermore, only the right middle incisors of young individuals were measured, so darker shades may not have featured. Given the rapid advancements in color determination equipment, further studies with updated technology are necessary.

## 5. Conclusions

The findings of this study indicate that the Trios3 Color intraoral scanner is superior to the visual method for determining tooth shade. To establish its application in clinical practice, the integration of intraoral scanners into university education is essential.

## Figures and Tables

**Table 1:** Measuring devices and specifications.

Device	TRIOS3 Color POD (T)	VITA Easyshade Advance 4.0 (V)	SpectroShade Micro (S)
Light source	LED	LED	LED
Radiation path	0°	0°	45°
Spectral resolution	Unknown	25 nm steps	10 nm steps

LED = light-emitting diode.

**Table 2:** Conversion table of VITA 3D-Master colors in CIE L*a*b* values, with the arithmetic mean of five values, measured with V.

VITA 3D-Master	L*	a*	b*
0M1	89.6	−0.1	7.1
0M2	84.3	0.3	8.5
0M3	85.7	−0.2	9.7
1M1	81.7	−0.1	11.7
1M2	80.9	−0.6	16.1
2L1.5	77.4	−0.3	15.5
2L2.5	78.1	−0.1	22.6
2M1	79.1	0.8	13.9
2M2	78.3	0.8	19.0
2M3	78.7	1.0	25.4
2R1.5	79.4	1.1	17.1
2R2.5	76.0	1.1	20.1
3L1.5	72.0	1.3	19.4
3L2.5	73.2	1.8	25.4
3M1	72.9	1.8	15.7
3M2	74.5	2.4	22.3
3M3	73.3	2.5	27.5
3R1.5	69.7	2.5	16.9
3R2.5	72.6	3.5	26.0
4L1.5	68.6	2.8	21.3
4L2.5	68.1	3.3	28.5
4M1	68.0	2.7	16.2
4M2	68.9	3.7	24.0
4M3	68.9	4.5	31.2
4R1.5	65.8	4.1	20.1
4R2.5	68.4	4.7	26.6
5M1	63.1	4.1	18.5
5M2	63.9	5.7	27.1
5M3	65.0	7.1	35.3

**Table 3:** Agreement rate following three to five direct repeat measurements.

	V	T	S
Agreement rate	75.8%	87.9%	89.9%

V = reference values of the point-measuring device; T = intraoral scanner; S = area-measuring device.

**Table 4:** Randomness of differences in agreement rates from Table 3.

Pearson Chi ^2^	V–T	V–S	T–S
*p*-value	0.010	0.002	0.651
Cramer’s V	0.124	0.146	0.032

V = reference values of the point-measuring device; T = intraoral scanner; S = area-measuring device.

**Table 5:** Color difference in ΔE as validity.

ΔE	V–T	V–S	V–DT
$\bar{x}$	3.8	8.3	7.4
SD	2.0	2.4	2.6
*p*-value	<0.001	<0.001	<0.001

V = reference values of the point-measuring device; T = intraoral scanner; S = area-measuring device; DT = dental technician; $\bar{x}$ = arithmetic mean; SD = standard deviation.

## Data Availability

The data presented in this study are available on reasonable request from the corresponding author.
